# Supplementary material for: Rhythmic coordination and ensemble dynamics in the hippocampal-prefrontal network during odor-place associative memory and decision making
Source: eLife. 2022 Dec 8;11:e79545. doi: 10.7554/eLife.79545 (PMC9799972; doi:10.7554/eLife.79545)
Supplement: Supplementary file 1. — Overall averages are indicated as mean ± s.e.m. [file elife-79545-supp1.docx]

| Animal | # of Sessions | Total CA1 | Active CA1 | CA1  pyr. | CA1  int. | CA1  task resp. | Total PFC | Active PFC | PFC  pyr. | PFC  int. | PFC  task resp. |
| --- | --- | --- | --- | --- | --- | --- | --- | --- | --- | --- | --- |
| 1 | 3 | 35.0 | 24.7 | 21.3 | 3.3 | 2.0 | 36.3 | 24.3 | 20.7 | 3.7 | 6.3 |
| 2 | 4 | 28.3 | 19.5 | 16.5 | 2.8 | 1.5 | 13.0 | 8.8 | 8.3 | 0.5 | 1.8 |
| 3 | 4 | 96.3 | 73.8 | 68.0 | 5.8 | 10.0 | 14.5 | 9.5 | 8.5 | 1.0 | 1.3 |
| 4 | 3 | 39.3 | 26.3 | 23.0 | 3.0 | 3.0 | 18.7 | 15.3 | 14.7 | 0.7 | 6.3 |
| 5 | 7 | 15.6 | 9.1 | 8.0 | 1.1 | 1.4 | 8.3 | 4.9 | 4.3 | 0.6 | 1.0 |
| 6 | 7 | 18.3 | 14.2 | 9.0 | 5.2 | 2.2 | 13.8 | 9.7 | 8.8 | 0.8 | 3.8 |
| 7 | 6 | 22.2 | 14.8 | 13.8 | 1.0 | 3.8 | 22.2 | 16.3 | 13.0 | 3.3 | 5.0 |
| 8 | 5 | 47.2 | 34.6 | 30.8 | 3.0 | 3.6 | 33.6 | 25.2 | 22.0 | 2.8 | 9.4 |
| Avg. |  | 37.8 ±  9.2 | 27.1  ± 7.2 | 23.8  ± 6.9 | 3.1  ± 0.6 | 3.4  ± 1.0 | 20.0 ±  3.6 | 14.2  ± 2.6 | 12.5  ± 2.2 | 1.7  ± 0.5 | 4.4  ± 1.0 |
